# Metastatic Adenocarcinoma of the Bladder Presenting as Malignant Pleural Effusion: A Rare Presentation of Bladder Adenocarcinoma

**DOI:** 10.7759/cureus.15152

**Published:** 2021-05-21

**Authors:** Rekha Ravikumar, Astrid Ross, Muhammad S Khan, Ghazal Khan, Shaili Desai

**Affiliations:** 1 Internal Medicine, Mercy Health, Toledo, USA; 2 Internal Medicine, University of Missouri, Kansas City, USA; 3 Oncology, Mercy Health, Toledo, USA

**Keywords:** malignant pleural effusion, metastatic adenocarcinoma of the bladder, rare presentation, brain and lung metastasis, non-urothelial bladder tumors, adenocarcinoma of unknown origin, extravesical bladder disease

## Abstract

Bladder cancers rarely are non-urothelial in origin. We present here, possibly the youngest case of a 35-year-old White female presenting with shortness of breath. She was found to have a malignant pleural effusion with unknown primary, eventually confirmed with genetic testing as metastatic adenocarcinoma of the urinary bladder with brain and lung metastasis. She was scheduled for palliative chemotherapy, however, passed away before it could be started. We highlight this rare case because of its unique presentation. Owing to similarity in receptors between adenocarcinoma and enteric cancer, similar chemotherapy regimens may be used for both. Unfortunately, treatment of metastatic disease remains highly controversial and needs to be studied further if there is an actual survival benefit to this or not.

## Introduction

Urothelial cancer represents 90% of newly diagnosed cases with non-urothelial cancers accounting for less than 5% [1,2]. Approximately 90% of non-urothelial cancers are epithelial in origin which include majorly adenocarcinoma, squamous cell carcinoma and small cell carcinoma with primary adenocarcinoma occurring in only 2% of total neoplasms [2-4]. There are several conditions associated with primary adenocarcinoma of the bladder (ACB) including schistosomiasis and bladder exstrophy [5,6]. It is the most common tumor after enterocystoplasty [7,8]. Although several atypical patterns of presentation of urothelial cancer have been described in literature such as involving the lymph nodes (90%), liver (47%), lung (45%) and the pleura (16%), ACB presenting as malignant pleural effusion has never been reported before [9]. We present here, possibly the youngest case of a 35-year-old White female, who was found to have a malignant pleural effusion with unknown primary, eventually confirmed with genetic testing as metastatic adenocarcinoma of the urinary bladder with brain and lung metastasis. This case report emphasizes the need to keep bladder cancer as a differential for malignant pleural effusion, prevent delay in diagnosis, assess the survival benefit of treating metastatic bladder cancer and initiate treatment early.

## Case presentation

A 35-year-old White female, non-smoker with no significant past medical history presented to the emergency department with gradual onset shortness of breath over the last two months.

**Figure 1:**
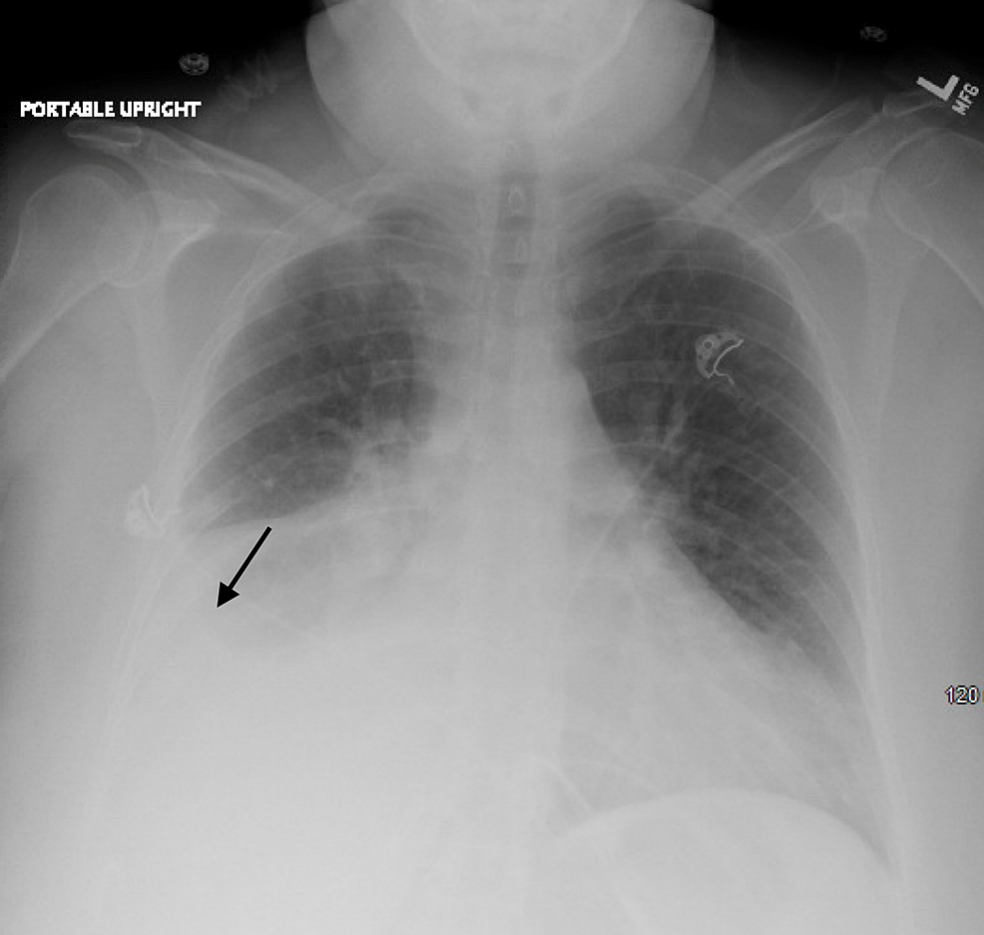
Portable chest X-ray on initial presentation showing massive right-sided pleural effusion with underlying airspace disease.

Initial laboratory work was unremarkable, chest X-ray (Figure 1) revealed a massive right-sided pleural effusion. She underwent thoracentesis and 6L of yellow hazy colored fluid was drained, microscopic analysis revealed exudative effusion with elevated nucleated cells 4,363/ul (>1000/ul is suggestive of exudative effusion) and red blood cells (RBC) 1,473/ul. Possible pleomorphic cells with hyperchromatic and multinucleated nuclei were visualized on direct exam, with negative lung markers TTF-7 (Thyroid transcription factor-7) and Napsin, making pulmonary primary less likely. Pathology was consistent with adenocarcinoma of unknown origin.

**Figure 2:**
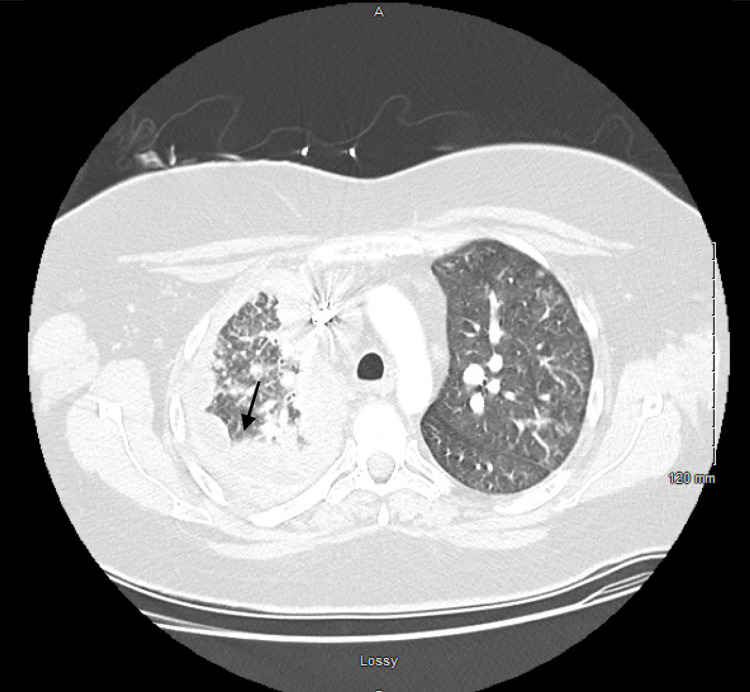
Computerized tomography scan of the chest with intravenous contrast showing moderate right-sided pleural effusion and near complete opacification of the hemithorax with minimal residual aeration in the right lung apex. Multiple nodular densities within the aerated left hemithorax.

**Figure 3:**
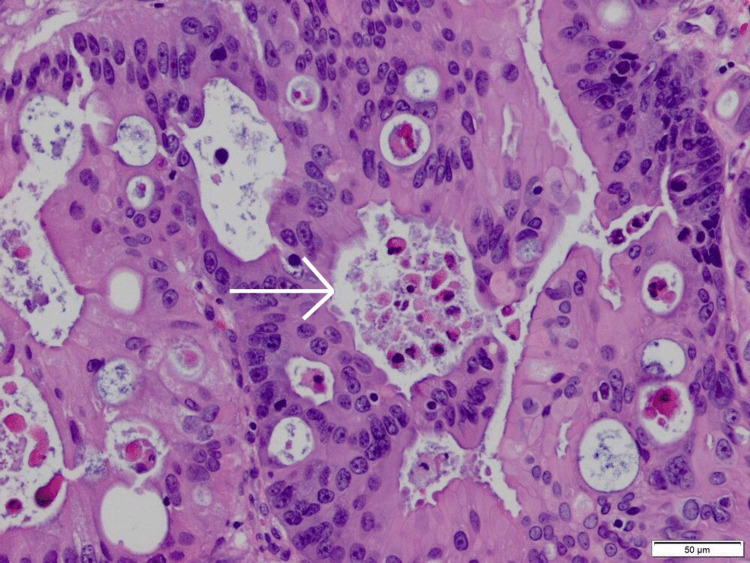
High resolution smear of biopsy of dome of the bladder illustrating large cells forming malignant glands with dirty necrosis (As pointed with white arrow).

**Figure 4:**
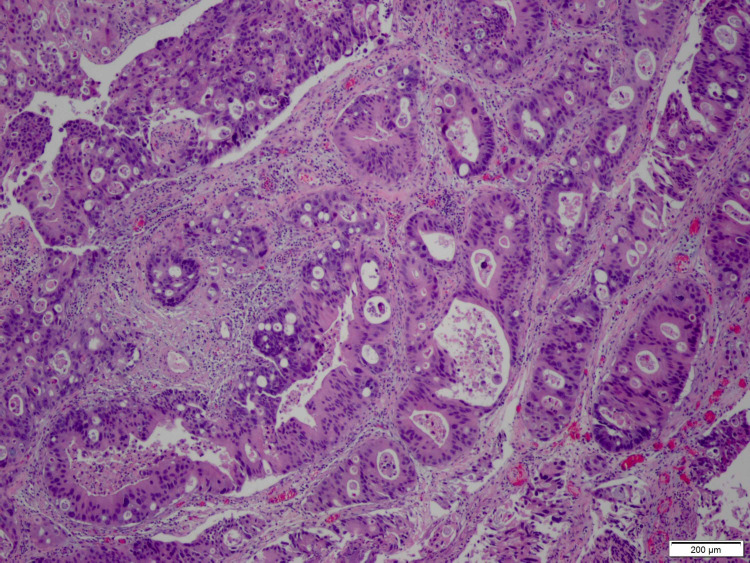
Low resolution smear of biopsy of dome of the bladder illustrating similar findings as in Figure 3.

Further workup was planned, with computerized tomography (CT) scan of the chest showing right-sided pleural effusion with minimal aeration (Figure 2), abdomen and pelvis revealing an infiltrative mass involving the dome of the bladder and adjacent small bowel loops. Ultrasound of the pelvis confirmed the bladder mass. Labs were normal except serum carcinoembryonic antigen was elevated at 9.1 ng/ml. This was followed by a positron emission tomography (PET) CT scan which revealed avid right lung circumferential pleural thickening with multiple mediastinal and paratracheal lymph nodes, possible iliacus, latissimus dorsi and L5 vertebrae involvement. Urology was consulted and transurethral resection of bladder tumor (TURBT) was planned and performed with excisional biopsies taken. This mass was eventually revealed as neoplastic likely the site of primary tumor (Figures 3, 4), an invasive carcinoma with glandular differentiation, with positive cytoplasmic staining with mostly tumors of enteric origin beta catenin, CDX2 (a member of homeobox transcription factor family), cytokeratin 20, and P53. P63, cytokeratin 5/6, cytokeratin 7, Estrogen receptor and GATA3 (another transcription factor) showed negative straining. Paired box gene 8 (PAX8) showed rare positive nuclear staining. The patient was subsequently discharged home. Plan was to evaluate for another possible primary in the colon upon follow-up.

However, she was readmitted after a few days while suffering from recurrent shortness of breath. Thoracentesis done this time drained 1L of cloudy fluid but failed to relieve her symptoms. Cardiothoracic surgery was consulted due to her history of previous pleural thickening on CT scan. They recommended surgical management and she underwent video-assisted thoracic surgery (VATS) with Talc pleurodesis. This resulted in significant improvement of her symptoms and she was discharged home with plans to follow up with hematology and oncology as outpatient.

**Figure 5:**
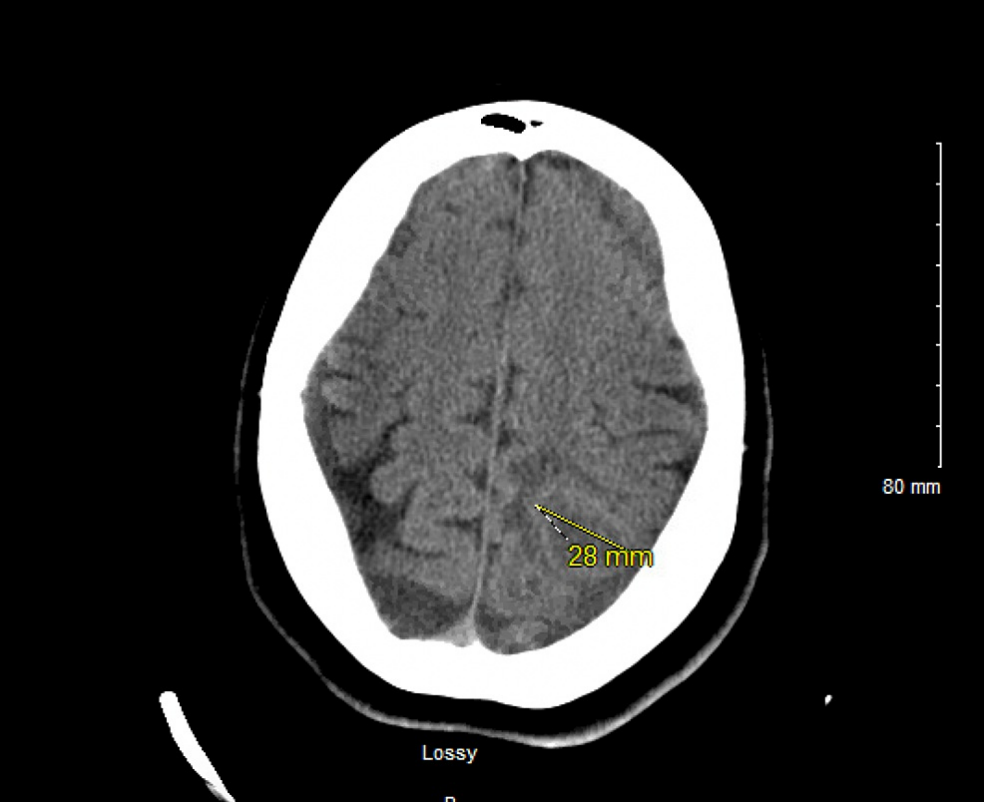
2.8 cm left parietal lobe mass with mild surrounding vasogenic edema as shown with an arrow sign. No significant mass effect or midline shift.

She presented again to the emergency department after a month, this time with seizures. CT scan of the brain revealed a new 2.8 cm left frontal mass (Figure 5), likely a metastatic lesion. Neurosurgery was consulted, the patient was started on intravenous (IV) levetiracetam with IV Methyl-prednisone. Eventually she underwent craniotomy with resection of the lesion which revealed malignant adenocarcinoma. On the same admission with colonoscopy to evaluate for possible primary, an 8-mm sessile polyp was excised in the transverse colon, however it was negative for invasive cancer. The patient was scheduled for outpatient chemotherapy pending testing for genetic mutation.

Genetic testing eventually revealed invasive adenocarcinoma with enteric differentiation. Cells stained positive for the KRAS oncogene and p53 mutation with gain of MYC oncogene. Decision was made to start her on mFOLFOX6 (Fluorouracil/Leucovorin/Oxaliplatin) regimen for a total of 12 cycles biweekly as outpatient. However, after she presented for first session of chemotherapy feeling very short of breath, she was sent to the emergency department instead after being deemed as not a candidate for chemotherapy due to poor general condition. On arrival, chest X-ray revealed a complete whiteout of the right lung, she was tachycardic and tachypneic and was sent for urgent thoracentesis under interventional radiology guidance. However, no significant fluid pockets could be identified and she was understood to have a trapped lung instead. Subsequently her condition worsened, she was transferred to the intensive care unit (ICU) and intubated. On day 2, she continued to require higher mechanical support with high end expiratory pressure and 100% of inspired oxygen. As per critical care, it would be extremely difficult to get her off ventilator support any time soon. Hematology and oncology at this time recommended palliation of care after extensive discussion with family. The patient’s code status was later changed to comfort care and she was terminally extubated and passed away.

## Discussion

Owing to the very low incidence of ACB, less is known about this tumor as compared to urothelial cancer (UCB). However, analysis of large-scale data using the Surveillance, Epidemiology, and End Results (SEER) database has sought to answer various questions regarding ACB outcomes and relation to prognostic factors such as stage, grade, metastasis and primary location of tumor [10-12].

Histologically, it can present as mucinous (colloid), papillary, signet-ring cell, clear-cell, or mixed subtypes [3,10]. Prognosis does not depend upon the histological outcome of the tumor [11]. However, some data suggests that the relatively poor prognosis with signet cell tumors might be due to their delayed presentation [12]. Tumors occurring at the dome of the bladder are also more likely to metastasize as compared to anterior, posterior and lateral wall tumors. This might be due to differential blood supply to the dome by the superior vesical vessels and is in concordance with our patient who presented with an already metastasized tumor with dome of the bladder as primary. Factors affecting tumorigenesis are believed to be development of metaplasia and chronic infection whereas alternative hypothesis includes the development of non-urothelial cancers from preexisting cancers. Tumor development from multipotent stem cells has also been shown to play a role [13].

The mean age at diagnosis for adenocarcinoma is 65.1 years with a greater proportion of males (62.8%) [12]. Our patient may yet be the youngest ever to present with adenocarcinoma in literature. Incidence was also greatest in non-Hispanic Caucasians as in ours followed by African Americans, Asian/Pacific islanders and American Indians.

Clinical presentation of non-urothelial bladder tumors is the same as urothelial bladder tumors (UBT), with painless hematuria the initial most common complaint, although sometimes they may have voiding problems such as frequency, urgency and dysuria [11]. Clinical presentation of metastatic tumors depends upon the systems involved; our patient presented with shortness of breath and seizures after involvement of the lungs and parietal lobe of the brain, respectively. Interestingly she did not have any gross hematuria till late thus resulting in late diagnosis.

Owing to relative rarity of the disease, no current guidelines exist to guide treatment. Surgery with or without lymph node dissection is usually the mainstay of treatment in limited disease regardless of histology [13]. A systemic review of literature revealed the majority of malignant urachal neoplasms to be well or moderately differentiated adenocarcinomas with extravesical spread [14]. Radical cystectomy remains the first line for surgical candidates [15,16] although other procedures such as local excision or partial cystectomies have also been performed. Our case involved a transurethral resection because the tumor did not encompass the whole bladder although she did have distant metastasis. Literature shows that patients with limited disease usually do have tumor excision, while more than two-thirds of patients with stage 4 disease undergo combined surgery and radiotherapy [15,16]. Prognosis correlates independently with both staging and grading. However, low-grade tumors with distal metastasis have extremely poor survival, in our case survival was barely three to four months. This is in concordance with previous observations that although nonurothelial tumors may be well differentiated than urothelial tumors at presentation, they are more likely to be extravesicular and have a poor prognosis.

Different case studies show that primary adenocarcinoma and colorectal cancers might be very similar in their tumor markers expression, morphological features and immunohistochemical characteristics which makes differentiation between the two challenging [15]. Recently, oxaliplatin or irinotecan in combination with either fluorouracil, bevacizumab or cetuximab have been found to be highly efficacious in treating both tumors [16-17]. FOLFOX regimen (fluorouracil, oxaliplatin plus leucovorin) has also been used as a frontline therapy due to similarity in cellular receptors [15,16]. In our case, the choice of chemotherapy was contemplated by two oncologists and FOLFOX regimen was eventually chosen due to the same reason. Unfortunately, our patient passed away before any treatment could be started [18,19]. The SEER study also demonstrated worse survival with distant metastasis at 8.1% at five years compared to 54.6% for localized disease [12]. Owing to very limited literature in extravesical bladder disease however, warrants further study on the use of adjuvant chemo and radiation therapy. Radiotherapy has been used in only a limited number of patients with UCBs to varying success [12]. Unfortunately, treatment for metastatic disease remains highly controversial and needs to be studied further if there is an actual survival benefit to this or not.

## Conclusions

Primary adenocarcinoma is a rare cause of bladder cancer and carries a poor prognosis than the more common urothelial tumors. This case report stresses the importance of including bladder adenocarcinoma as a differential for unilateral malignant pleural effusion. When diagnosed early, non-metastatic non-urothelial bladder carcinoma can be surgically resected. Owing to similarity in receptors between adenocarcinoma and enteric cancer, similar chemotherapy regimens may be used for both. Unfortunately, treatment for metastatic disease remains highly controversial and needs to be studied further if there is an actual survival benefit to this or not.
